# Prevalence of Visual Symptoms and Deficits Among Inpatients with Acquired Brain Injury

**DOI:** 10.3390/neurolint18080153

**Published:** 2026-08-19

**Authors:** Camilla Biering Lundquist, Lars Evald, Simon Svanborg Kjeldsen, Lis Viborg, Rasmus Langelund Jørgensen, Jesper Fabricius

**Affiliations:** 1 Hammel Neurorehabilitation Centre and University Research Clinic, 8450 Hammel, Denmark; lareva@onerm.dk (L.E.); jespmr@onerm.dk (J.F.); 2 Department of Clinical Medicine, Aarhus University, 8200 Aarhus, Denmark

**Keywords:** visual deficits, vision screening, vision-related impairments, ocular motility, visual field loss, patient-reported visual deficits, acquired brain injury, stroke, rehabilitation

## Abstract

Objectives: To determine the prevalence of visual deficits among inpatients with acquired brain injury, to examine the agreement between objective screenings and patient-reported visual deficits, and to investigate patient characteristics associated with visual deficits. Methods: This cross-sectional study included data on screened and patient-reported visual deficits in patients with acquired brain injury admitted for inpatient rehabilitation. The screenings were conducted by occupational therapists. Visual acuity and visual field were assessed in 2014–2023, and complemented by assessment of ocular motility and patient-reported deficits in 2021–2023. Predictive values were calculated for the agreement between screening results and patient-reported symptoms. Logistic regression models were applied to investigate the association between clinical characteristics and visual deficits. Results: 2814 patients were screened, 65% were men, the median age was 64 years, and the most common diagnosis was stroke (66%). In screened patients 32% had reduced visual acuity, 18% had ocular motility deficits, and 15% had visual field deficits. Of 975 patients providing self-reports, 38% reported vision problems. A total of 28% of patients who did not report visual deficits, had visual deficits in the objective screening. Lower functional level was associated with greater odds of having any visual deficits. Traumatic and anoxic brain injuries were associated with lower odds of visual field deficits compared with stroke. Conclusions: The high proportion of patients with visual deficits underlines that these are common consequences following acquired brain injury. Objective screening and patient-reported deficits offer overlapping yet distinct insights into visual deficits. Knowledge of clinical characteristics associated with visual deficits may help recognize high-risk subgroups.

## 1. Introduction

Visual deficits are common after acquired brain injury (ABI) [1,2], leading to challenges such as difficulty in reading, increased risk of falls, greater utilization of community services, and a higher incidence of depression and social isolation [3,4,5]. In addition, visual deficits are associated with decreased participation in meaningful activities and reduced quality of life [6]. From a patient perspective, visual changes often disrupt life in profound ways, yet many report that these problems receive insufficient attention in the healthcare system [7].

Visual deficits are often categorized into four categories: reduced visual acuity, visual field loss, ocular motility abnormalities, and visual perception deficits [1,8]. Unrecognized visual deficits can have a major impact on the patient’s recovery process [9], and knowledge about the prevalence and distribution of visual deficit is essential for applying the appropriate precautions and interventions. In line with this, several recommendations advocate standardized screening for visual changes in individuals with ABI, in settings where ophthalmologists or orthoptists are not readily available [1,9,10,11,12].

Although multiple studies have examined the occurrence of visual deficits, reported prevalence estimates vary widely [8,13,14,15,16]. This heterogeneity reflects differences in subpopulations (etiology, stage post injury), the screening or assessment applied, the range of visual domains assessed, and the interval from injury to screening. A large multicenter study by Rowe et al. reports the presence of central visual impairment in 43.9%, visual field loss in 25.6%, ocular motility abnormalities in 44.3%, visual inattention in 26.2%, and visual perception deficits in 4.9% of stroke survivors. Additionally, these deficits occurred as new onset (incidence) in 29.4%, 24.7%, 39.3%, 26.2%, and 4.7%, respectively [8]. In a study conducted on 323 stroke patients in 14 acute hospitals, Rowe et al. also found that 68% had eye alignment/movement impairment, 49% had visual field impairment, 26.5% had reduced visual acuity, and 20.5% had perceptual difficulties [16]. Schoff et al. found that 101 (46%) of examined patients with ABI experienced visual deficits [13], while Norup et al. reported that 84 (24.1%) patients in the acute stage of stroke had visual or visual–attentional problems [14]. Notably, in some of the above-mentioned studies only patients with already suspected visual deficits were included, limiting inference about the overall burden in broader rehabilitation cohorts [13,14,15,16]. Moreover, visual deficits are not always overt [9,16], and compromised visual function may only be recognized when tasks requiring visual input are affected [1]. As a result, visual deficits can remain unrecognized by both patients and staff in a rehabilitation setting [8,15].

The above-mentioned factors highlight the need for large-scale studies that determine the prevalence of visual deficits in a clinically representative population of patients with acquired brain injury, irrespective of whether visual deficits are suspected at inclusion. Such studies can provide a more accurate understanding of the burden of visual impairment and support evidence-based decisions related to screening practices, and the rehabilitation trajectory. In addition, the relationship between objectively identified visual deficits and patient reported visual symptoms remains insufficiently characterized. It is hypothesized that different aspects of visual deficits will be identified by the two different methods. For instance, patients with reduced insights after ABI may be unaware of their visual deficits, and these may be captured in an objective screening only. Also, little is known about clinical characteristics associated with the presence of visual impairments in inpatient neurorehabilitation populations. Identifying such associations may help clinicians recognize high-risk subgroups, prioritize specialist assessments, and tailor rehabilitation strategies.

The primary objective of this study was to determine the prevalence of visual deficits in a large population of patients with ABI admitted for inpatient neurorehabilitation. Secondary objectives were (1) to examine the agreement between objective screening findings and patient-reported visual deficits, and (2) to investigate patient characteristics associated with visual deficits.

## 2. Materials and Methods

### 2.1. Study Design

This is a cross-sectional study. Standardized vision screenings were conducted, and the results were collected from electronic medical records.

### 2.2. Setting and Population

The study was carried out at an inpatient neurorehabilitation hospital in Denmark, with 115 beds across 10 wards. Patients were in the early-to-late post-acute phase following ABI and required comprehensive neurorehabilitation. Records assessed for eligibility were from patients with stroke, traumatic, or anoxic brain injury admitted between 1 January 2014, and 31 December 2023. The inclusion criteria were objective vision screening and/or patient reports of visual symptoms performed within six months of injury.

### 2.3. Vision Screening

A local clinical guideline prescribes a mandatory screening for visual deficits within 14 days of admission to neurorehabilitation. Occupational therapists are responsible for conducting vision screenings, which is common practice [17,18,19]. Based on the screening findings and observations during rehabilitation, the medical doctor and the patient’s interdisciplinary team will evaluate whether a more detailed vision examination is necessary. This evaluation may lead to a referral to an ophthalmologist [17,18].

**Figure 1:**
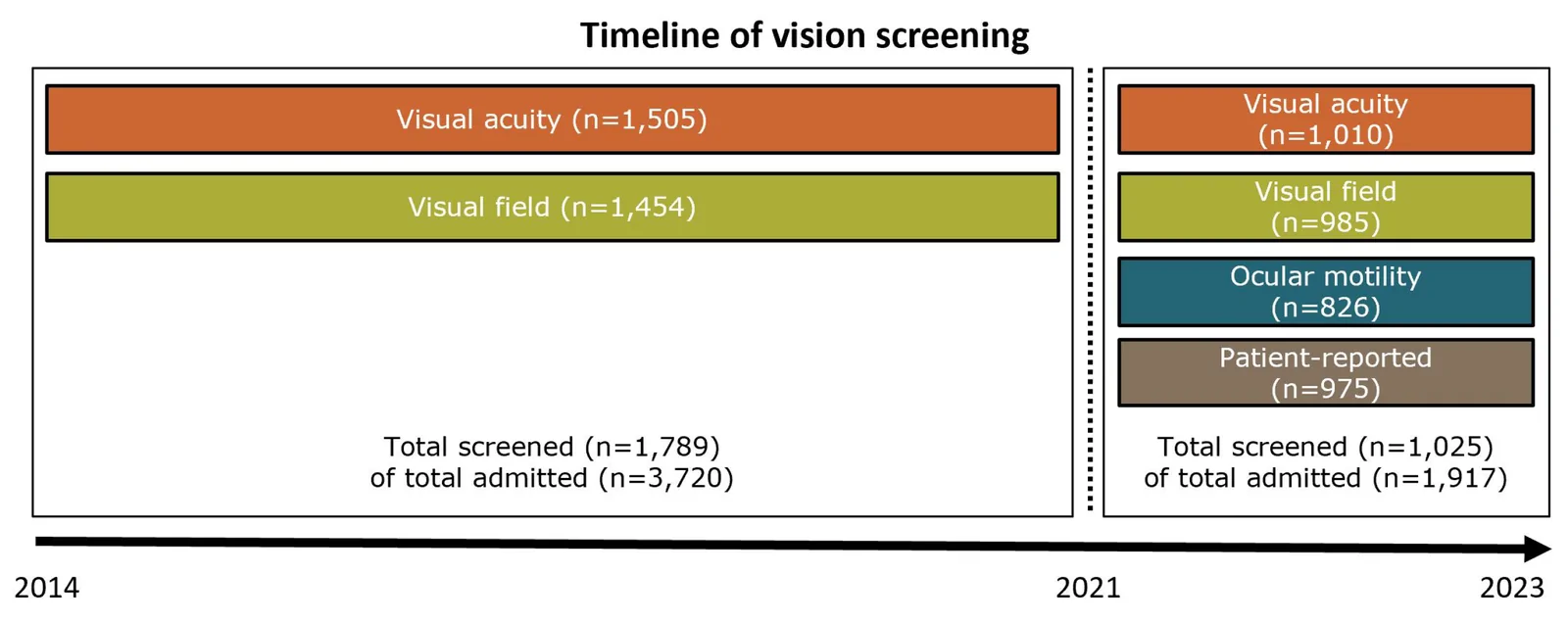
Timeline of visual deficits included in the vision screening and number of patients screened for each deficit. Note: Two different methods were used to assess visual field deficits in 2014–2020 and 2021–2023.

From 2014 to 2020, vision screening consisted of a screening for reduced visual acuity and a screening for visual field deficits (Figure 1). In 2021, the screening process was expanded to include ocular motility deficits and a patient-reported evaluation of vision deficits (see Figure 1).

Visual acuity was screened using the Snellen Eye Chart, which evaluates the ability to discern letters or characters of high contrast at decreasing sizes, relying on central vision. Patients were instructed to use their glasses if required. Reduced visual acuity was defined as being less or equivalent to 6/18 on the Snellen Eye Chart, corresponding to the World Health Organization levels for reduced visual acuity [20].

The visual field was screened with one eye covered. From 2014 to 2020, the screening guideline instructed the examiner to stand behind the patient and slowly move a pen from a 90-degree angle into the patient’s visual field until the patient could see the pen while looking straight ahead. Visual field loss was defined as having less than 10 degrees of vision in either direction. From 2021 onward, the guideline for screening visual field deficits was changed to a confrontation test, with the examiner in front of the patient. The patient was instructed to look straight ahead while the examiner performed finger movements, bringing his or her hands into the patient’s visual field from the lateral side of each quadrant. For the present study, visual field loss was defined as the loss of part of the central and/or peripheral field of vision, categorized as cortical blindness, bitemporal hemianopia, homonymous hemianopia, and other visual field loss.

Ocular motility was examined binocularly by tracing an “H” pattern in front of the patient while the patient held head still. Ocular motility abnormalities were categorized as unparalleled eye axis, restricted eye movement, and tendency for eyelid closure.

In the patient-reported part, patients were asked if they used glasses or contact lenses and if they had experienced new vision problems, double or blurred vision, altered light sensitivity, reading problems, and/or changes in the perception of color.

### 2.4. Data and Data Analysis

Data on vision screening and information on age, sex (at birth), diagnosis, time from injury to admission, length of stay at the hospital, and Functional Independence Measure (FIM) [21] at admission, were extracted from medical records. FIM is an ordinal score ranging from 18 to 126, with higher scores representing a higher degree of independence in function and activities of daily living [21].

Demographic and clinical characteristics were presented using descriptive statistics. Variables on continuous scales were summarized by median and interquartile range (IQR) and categorical variables by numbers and percentages. The Wilcoxon rank-sum test or Chi^2^ test was used to test whether the patients screened differed in demographic and clinical characteristics from patients who were not screened. The overall prevalence of visual deficits was reported, as was the prevalence within each of the specific visual deficit categories (visual acuity deficits, visual field deficits, ocular motility deficits, and patient-reported deficits). As the screening for visual field deficits was changed in 2021, the prevalence of visual field deficits found in each time period was also reported separately.

Predictive values were presented for the agreement between the vision screening and patient-reported visual deficits for patients with complete data for both the vision screening and the patient-reported visual deficits. Multiple adjusted logistic regression analyses were applied to investigate the association between patients characteristics (age, diagnosis, weeks since injury, and FIM score) and visual deficits (reduced visual acuity, visual field deficit, and ocular motility deficits). Analyses were performed with STATA V18.

## 3. Results

From 1 January 2014 to 31 December 2023, a total of 5637 patients with stroke, TBI, or anoxic brain injury were admitted for neurorehabilitation within six months of their injury. A total of 2814 (50%) patients were screened within 6 months of injury (Table 1). The median age was 64 years (IQR 54–72), 65% were men and the most frequent diagnosis was stroke (see Table 1). The median FIM score was 69 (IQR range 29–101) on admission, reflecting a wide variety of independence in motor and cognitive function. Non-screened patients were significantly younger than screened patients, more had ischemic stroke while less had heamorrhagic stroke, SAH or TBI. Length of rehabilitation stay was below 14 days in significantly more non-screened patients (Table 1). Screening completeness varied between the 10 wards, from 27% to 81%.

**Figure 2:**
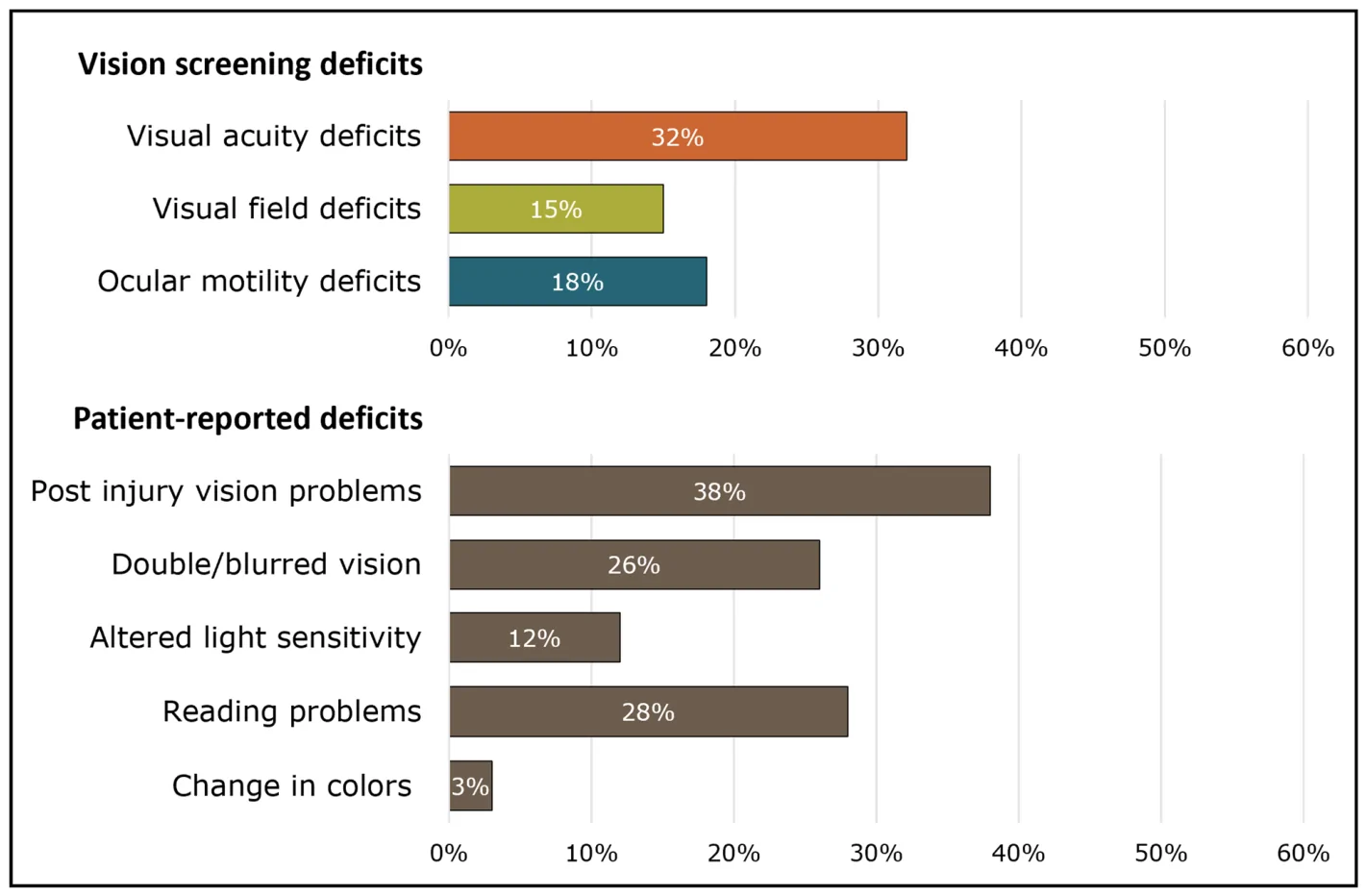
Prevalence of visual deficits in patients with acquired brain injury.

Table 2 and Figure 2 presents screening results and patient-reported visual deficits for all patients according to the main diagnostic groups. Reduced visual acuity affected 808 (32%) patients, 149 (18%) had an ocular motility deficit, and 376 (15%) had visual field deficits. The screening for visual field deficits was changed in 2021 and 172 (12%) and 204 (21%) had visual field deficits in the periods 2014–2020 and 2021–2023, respectively.

In the patient-report part (Table 2), 371 (38%) patients reported having post-injury vision problems. The most frequently reported issues were reading problems and blurred/double vision, which were reported by 264 (28%) and 251 (26%) of patients, respectively.

**Figure 3:**
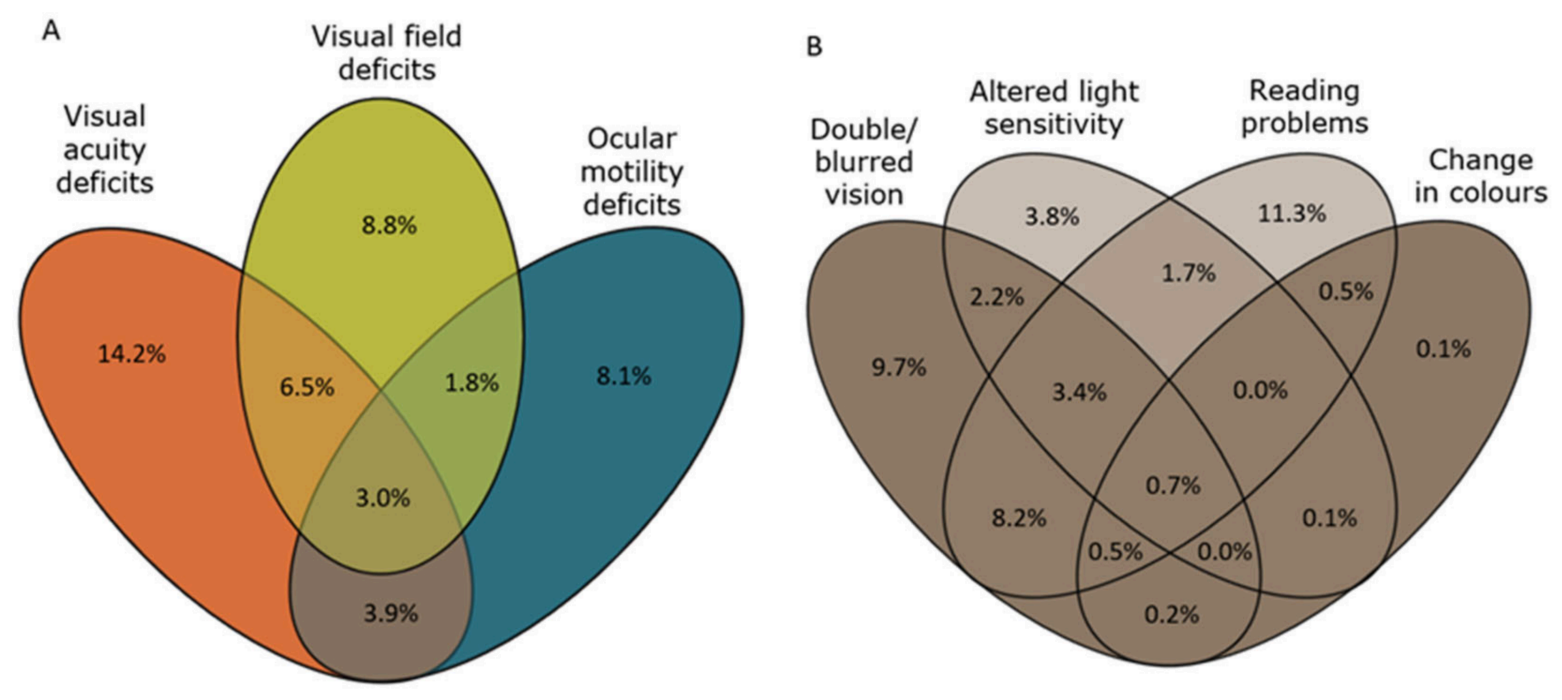
Prevalence of combinations of screened and patient-reported visual deficits. Legend Figure 3: (**A**) Venn diagram depicting prevalences of combinations of visual deficits among the 2021–2023 sample with screening data on all three assessments (*n* = 787), of which 364 (46.3%) had some kind of visual deficit. (**B**) Venn diagram depicting prevalences of combinations of patient-reported visual deficits (altered light sensitivity, change in colors, reading problems and double/blurred vision) among the 2021–2023 sample with complete patient-reported outcomes (*n* = 877), of which 372 (42.4%) reported some kind of visual deficit.

Some patients experienced more than one visual deficit, and Figure 3 illustrates the combination of prevalent deficits identified in a subpopulation of patients with all three assessments. The most common combination was reduced visual acuity combined with a visual field deficit, which was seen in 6.5%. In the patient-reported part, the most common combination was double/blurred vision and reading problems which was seen in 8.2%.

The agreement between screened and patient-reported visual deficits was assessed for patients screened in 2021–2023, in 975 patients with data on both outcomes. The positive predictive value in Table 3 shows that only 49% of patients who reported new visual deficits had findings of visual deficits in the screening. The negative predictive value shows that 72% of patients who did not report new visual deficits had no deficits in the screening. However, this also means that 28% of patients who did not report any new visual deficits, in fact, had visual deficits identified when screened.

In Table 4, the associations between patient characteristics and visual deficits are displayed. As can be seen, there was an anticipated distinct age gradient for the association with visual acuity, showing that visual acuity deficits are more common in older age. It is also evident that visual acuity deficits are less common in anoxic brain injury patients compared with stroke patients, and visual field deficits are less common in TBI and anoxic brain injury patients compared with stroke patients. Lastly, there was an anticipated distinct gradient for the association between functional level (FIM score as a proxy for injury severity) and all visual deficits, showing that a lower functional level post injury was associated with higher odds of having visual deficits.

## 4. Discussion

This large cross-sectional study establishes a high prevalence of visual deficits among patients undergoing inpatient neurorehabilitation after ABI. It was found that 32% of the patients had visual acuity deficits, 18% had ocular motility deficits, and 15% had visual field deficits. In the patient-report part, 38% of patients reported vision problems. A substantial proportion of patients suffered multiple concurrent visual problems, highlighting the clinical complexity of vision-related impairments in this population.

The vision screening protocol was considerably changed during the study period. The change in screening procedure in 2021 to a more detailed confrontational test for visual field deficits resulted in more patients identified with visual field deficits in 2021–2023. Additionally, the expansion of the screening procedure to include ocular motility deficits and a patient-reported evaluation of visual deficits resulted in more deficits being identified. In the present study, the prevalence of ocular motility and visual field deficits was generally lower than previously reported [8,14,15,22]. However, comparing the prevalence of visual deficits between studies is challenging for various reasons. First, several different vision screening tools exist, each focusing on different aspects of visual deficits [1,10,17,23], and there is no consensus on which screening method to use [17,24]. In the present study, confrontational vision screening and not a comprehensive examination was conducted. Thus, minor deficits may have gone unrecognized. Second, compared to studies performed in acute settings, the screening is performed later post-injury, and several deficits, especially visual acuity, ocular motility abnormalities, and visual perception deficits, are known to recover spontaneously over the first weeks and months [8]. Third, the population in the present study spans a broad spectrum of patients with ABI, while other studies have included only patients with stroke [2,8] or only patients with suspected visual impairment [13,14,15,16]. In patients with suspected visual impairment, Suchoff et al. found deficits in 62 (39%) of patients with TBI and in 40 (67%) of patients with stroke [13].

Some studies report that homonymous hemianopia is the most common visual deficit [13,14]. However, we confirm other previous findings, showing that ocular motility deficits occur more frequently [8,15]. Consistent with a study by Rowe et al. [2], many patients in the present study experienced more than one visual problem.

The partial agreement between objective screening results and the patient-reported symptoms aligns with previous studies showing that patients are often unaware of their visual deficits and that the presence of visual deficits is underestimated in subjective reports [8,15,25,26]. Almost 40% of patients with stroke-related visual deficits do not report visual symptoms during acute care [26]. The high proportion of patients unaware of their visual deficits highlights the importance of objective screening, to identify new deficits that would otherwise have gone unrecognized. Furthermore, in patients with complex symptomatology, a neuropsychological examination may help distinguish the difficulties and uncover visual attention and perception disorders [8].

The strengths of this study are the large sample size, along with standardized screening procedures. Unlike most studies, the study reflects a consecutive, clinically representative population of patients with acquired brain injury, irrespective of whether visual deficits were suspected at inclusion. The prevalence of visual deficits observed are likely comparable to what would be found in other rehabilitation settings with similar patient populations.

Several limitations of the study warrant consideration. Although vision screening was mandated, 2823 (50%) patients were not screened, a proportion comparable to a study by Rowe et al., were 48.4% of patients could not be accessed early after stroke because they were medically unwell, lacking sufficient attention or cognition, or suffering from fatigue [2]. In our study the specific reasons for missed screenings remain unclear. Screening rates varied substantially across wards, from 27% to 81%, indicating inconsistent implementation of screening procedures. Furthermore, as noted by Rowe et al., patients with the most severe cognitive or physical impairments are unlikely to participate in vision screening. Additionally, data show that significantly more non-screened patients had a rehabilitation stay shorter than 14 days, leading to discharge before the mandatory screening deadline. Collectively, these factors may result in underrepresentation of both severely affected and mildly disabled patients among those screened, introducing potential structural selection bias.

The vision screening is performed by occupational therapists, which is common practice [17,18,19]. Occupational therapists do not have formal education in vision deficits in their education. However, the screening is fairly simple, and it is argued that systematic errors in the screening are unlikely. At most, there may be a small degree of random error, which does not introduce information bias.

As in a previous study [1] it remains unclear how many of the identified deficits in the present study were caused by the recent ABI and how many were pre-existing conditions. Thus, reduced visual acuity may be attributed to pre-existing conditions such as age-related macular degeneration or glaucoma [4,22,27]. Nevertheless, new onset of reduced visual acuity can also arise from injury-related disruption of the visual pathway. The retina receives its arterial blood supply from the central retinal artery, and adequate perfusion is essential for maintaining central vision [2]. It is therefore plausible that reduced central vision following stroke or other ABI may, at least in part, reflect impaired vascular perfusion and relative ischemia within the anterior visual pathway [8]. In the present study, we did not have information on the specific location of the injury. Still, damage to specific brain areas results in different levels of vision-related functional impairments [22,28] and it would have been relevant to examine how location of the injury is associated with visual deficits. Regarding the generalizability of the findings to other post-acute rehabilitation hospitals, the detailed Table 1 allows readers to compare the screened populations to those of other post-acute rehabilitation settings.

## 5. Conclusions

This large cross-sectional study established that visual deficits are common among patients with ABI hospitalized for rehabilitation, with a gradient showing that lower functional level is associated with greater odds of vision deficits. Nearly one-third of patients who did not report visual difficulties were found to have objective vision impairments.

The implications of the findings are that early identification of vision deficits along with information of risk stratification during rehabilitation helps clinicians detect high-risk groups and allows for interventions targeting the specific vision deficits. Furthermore, it should be recognized that objective vision screening and patient-reported evaluations offer complimentary insight into vision deficits.

Presentation of patient characteristics makes it possible to assess the generalizability of findings to other post-acute settings.

## Figures and Tables

**Table 1:** Characteristics of patients with a vision screening within six months of injury.

Characteristics	Screened (*n* = 2814)	Not Screened (*n* = 2823)	*p*-Value
Age, median (IQR)	64 (54–72)	59 (49–69)	<0.001 *
Women ^1^, *n* (%)	973 (35%)	1024 (36%)	0.191
Men ^1^, *n* (%)	1841 (65%)	1799 (64%)	
Diagnosis			
- Ischemic stroke, *n* (%)	1297 (46%)	1490 (53%)	<0.001 *
- Hemorrhagic stroke, *n* (%)	568 (20%)	465 (16%)	<0.001 *
- SAH, *n* (%)	268 (10%)	208 (7%)	0.004 *
- Stroke NOS, *n* (%)	133 (5%)	157 (6%)	0.156
- TBI, *n* (%)	405 (14%)	330 (12%)	0.003 *
- Anoxic, *n* (%)	143 (5%)	173 (6%)	0.088
Days from injury until admission, *n* (%)	18 (9–35)	17 (7–41)	0.068
FIM, on admission, median (IQR)	69 (29–101)	76 (31–103)	0.063
Length of stay < 14 days, *n* (%)	136 (5%)	381 (14%)	<0.001 *

Abbreviations: SAH = subarachnoid hemorrhage; NOS = not otherwise specified; TBI = traumatic brain injury; FIM = functional independence measure. ^1^ Sex assigned at birth. * Statistically significant difference.

**Table 2:** Screened and patient-reported visual deficits in patients with acquired brain injury.

Visual Acuity	Stroke (*n* = 2047)	TBI (*n* = 347)	Anoxic (*n* = 118)	Total (*n* = 2512)
Reduced (6/>12), one eye	380 (24%)	57 (16%)	11 (9%)	**448 (18%)**
Reduced (6/>12), both eyes	306 (15%)	40 (12%)	14 (12%)	**360 (14%)**
**Patients with reduced acuity (6/>12)**	686 (34%)	97 (28%)	25 (21%)	**808 (32%)**
**Visual field deficit**	Stroke (*n* = 2000)	TBI (*n* = 325)	Anoxic (*n* = 116)	**Total (*n* = 2445)**
Cortical blindness	17 (1%)	3 (1%)	1 (1%)	**21 (1%)**
Bitemporal Hemianopia	9 (1%)	1 (1%)	0 (0%)	**10 (1%)**
Homonymous Hemianopia	97 (5%)	4 (1%)	2 (2%)	**103 (4%)**
Other visual field loss *	216 (10%)	19 (5%)	7 (6%)	**242 (9%)**
**Patients with ≥ 1 visual field deficit**	339 (17%)	27 (8%)	10 (9%)	**376 (15%)**
**Ocular motility abnormalities**	Stroke (*n* = 696)	TBI (*n* = 91)	Anoxic (*n* = 39)	**Total (*n* = 826)**
Unparalleled eye axis	59 (9%)	7 (8%)	2 (5%)	**68 (8%)**
Restricted eye movement	100 (15%)	10 (11%)	4 (11%)	**114 (14%)**
Tendency for eyelid closure	26 (4%)	7 (8%)	1 (3%)	**34 (4%)**
**Patients with ≥1 ocular abnormality**	128 (18%)	15 (16%)	6 (15%)	**149 (18%)**
**Patient-reported visual deficits**	Stroke (*n* = 825)	TBI (*n* = 103)	Anoxic (*n* = 47)	**Total (*n* = 975)**
Post-injury vision problems	327 (40%)	33 (32%)	11 (23%)	**371 (38%)**
Double/blurred vision	214 (26%)	28 (26%)	9 (19%)	**251 (26%)**
Altered light sensitivity	102 (12%)	12 (11%)	3 (6%)	**117 (12%)**
Reading problems	231 (29%)	24 (23%)	9 (19%)	**264 (28%)**
Change in colors	22 (3%)	3 (3%)	0 (0%)	**25 (3%)**
Patients with ≥1 patient-reported deficit	447 (55%)	54 (53%)	16 (34%)	**517 (54%)**

* Includes visual field loss in one quadrant and visual field loss in one eye only. Based on data collected from 2021 to 2023 on 985 patients, a total of 44 (4%) of patients had quadrantanopia in only one of the four quadrants. This was evenly distributed with 1% of screened patients having hemianopia in each of the four quadrants, respectively.

**Table 3:** Agreement between screened and patient-reported visual deficits.

	Screened Visual Deficits (Visual Acuity Excluded)		
Patient-reported visual deficits	Yes	No	Total
Yes	(A) 180 (49%)	(B) 191 (51%)	371 (38%)
No	(C) 171 (28%)	(D) 433 (72%)	604 (62%)
Total	351 (36%)	624 (64%)	975 (100%)

Data for the period 2021–2023 on 975 patients with data on both outcomes. (A) Positive predictive value; (B) false positive; (C) false negative; (D) negative predictive value.

**Table 4:** Associations between patient characteristics and visual deficits in subjects with acquired brain injury admitted for inpatient rehabilitation.

	Reduced Visual Acuity (*n* = 2472)		Visual Field Deficit (*n* = 2400)		Ocular Motility Deficits (*n* = 804)	
	Cases/Subjects	Adjusted Odds Ratio	Cases/Subjects	Adjusted Odds Ratio	Cases/Subjects	Adjusted Odds Ratio
Age						
0–18	16/80	0.38 (0.21; 0.69)	5/75	0.47 (0.18; 1.22)	3/18	0.83 (0.21; 3.27)
19–44	92/361	0.52 (0.39; 0.69)	46/352	0.80 (0.55; 1.17)	21/105	0.87 (0.48; 1.56)
45–64	320/1145	0.56 (0.46; 0.68)	168/1112	0.83 (0.65; 1.07)	54/348	0.59 (0.39; 0.90)
≥65	369/886	1	153/861	1	71/333	1
Diagnosis						
Stroke	680/2019	1	336/1967	1	128/681	1
TBI	94/341	0.85 (0.64; 1.13)	26/321	0.40 (0.26; 0.62)	15/88	0.75 (0.39; 1.42)
Anoxic	23/112	0.60 (0.37; 0.97)	10/112	0.44 (0.23; 0.88)	6/35	0.91 (0.35; 2.39)
Weeks since injury						
0–2	380/1223	1	168/1222	1	58/443	1
2–4	196/632	1.00 (0.79; 1.26)	104/598	1.32 (0.98; 1.76)	43/203	1.48 (0.89; 2.35)
4–8	172/469	1.22 (0.95; 1.57)	80/441	1.25 (0.90; 1.73)	41/128	2.32 (1.35; 3.96)
>8	49/148	1.18 (0.80; 1.73)	20/139	1.12 (0.67; 1.89)	7/30	1.77 (0.69; 4.54)
FIM						
18	60/131	3.10 (2.08; 4.60)	32/118	3.23 (2.00; 5.22)	11/32	3.58 (1.53; 8.37)
19–36	193/492	1.94 (1.51; 2.51)	104/447	2.12 (1.53; 2.92)	38/100	3.96 (2.23; 7.05)
37–90	325/892	1.69 (1.37; 2.09)	130/878	1.25 (0.95; 1.66)	62/300	1.98 (1.26; 3.10)
91–126	219/957	1	106/957	1	38/372	1

Abbreviations: TBI = traumatic brain injury; FIM = functional independence measure. Odds ratios are mutually adjusted for the other explanatory variables in the table.

## Data Availability

The raw data supporting the conclusions of this article will be made available by the authors on request.
